# CONTExT-RA: a cross-sectional study evaluating disease activity, quality of life and the socio-demographic profile of Irish patients with rheumatoid arthritis

**DOI:** 10.1093/rap/rkae132

**Published:** 2024-11-06

**Authors:** Grainne Murphy, Killian O’Rourke, Angela Camon, David Kane, Finbar O’Shea, Richard Conway, Claire Sheehy, Moneeb Saddiq, Deirdre Moran

**Affiliations:** Department of Rheumatology, Cork University Hospital, Cork, Ireland; Department of Rheumatology, Midlands Regional Hospital, Tullamore, Ireland; Department of Rheumatology, Midlands Regional Hospital, Tullamore, Ireland; Department of Rheumatology, Tallaght Hospital, Dublin, Ireland; Department of Rheumatology, St James’s Hospital, Dublin, Ireland; Department of Rheumatology, St James’s Hospital, Dublin, Ireland; Department of Rheumatology, University Hospital Waterford, Waterford, Ireland; O4 Research Limited, Belfast, UK; AbbVie Limited, Dublin, Ireland

**Keywords:** RA, disease activity, Irish population, DMARDs, CDAI, quality of life

## Abstract

**Objectives:**

CONTExT-RA is a cross-sectional, non-interventional multicentre study which enrolled patients diagnosed with RA and receiving DMARD treatment in a secondary care setting. The study evaluated disease control and associated disease burden amongst this Irish population.

**Methods:**

Patients with RA attending six Irish rheumatology centres were invited to participate. Each consented patient attended a single routine study visit. Disease activity was assessed using Clinical Disease Activity Index (CDAI). The primary endpoint was EuroQol-5 dimensions (EQ-5D-5L) stratified by CDAI, compared using a non-parametric Wilcoxon Rank-Sum test.

**Results:**

130 patients were included. Using CDAI, 34 (26.2%) patients were in clinical remission (CR), 42 (32.3%) had low disease activity (LDA), 41 (31.5%) had moderate disease activity (MDA) and 13 (10.0%) had high disease activity (HDA). QoL (EQ-5D-5L index (median)) scores were significantly (*P* < 0.001) greater for patients in CR or CR/LDA than for those with MDA/HDA, 0.866 (0.920), 0.777 (0.822) vs 0.578 (0.691), respectively. Patients in CR reported higher levels of work productivity, mean (s.d.) rating of 1.7 (2.52) vs those in MDA/HDA of 4.2 (3.28) (higher rating indicates greater impairment). Similar findings were observed for non-work-related activities.

**Conclusion:**

Disease control for many patients with RA, treated in secondary care in Ireland, is sub-optimal with only 1 in 4 in CDAI remission. The impact of poor disease control on QoL is significant, and the superior outcomes for patients in CR provide compelling evidence that by achieving greater disease control, the burden of disease on patients can be greatly reduced.

Key messages

- Disease control for many patients with RA treated in Irish secondary care is not optimal.
- The observed impact of poor disease control is significant and wide-ranging.
- Outcomes for patients in remission evidence the value of striving for greater disease control.

## Introduction

Although estimates vary, a recent meta-analysis of data from 67 published studies reported the global RA prevalence as 0.46% [1]. Disease prevalence and incidence are increasing, with reports suggesting these are highest in North America and Western Europe [2]. The National Medicines Information Centre reports approximately 1% of the population is affected, and Arthritis Ireland estimates 45 000 Irish patients are living with RA with 2000 new cases annually [3, 4].

As one of the most common autoimmune diseases and the most common autoimmune arthritis [5, 6], RA is a chronic inflammatory disease which, if not controlled, may result in severe and progressive articular injury, loss of function/productivity, deterioration in quality of life (QoL) and increased morbidity/mortality [7–10]. Patients with RA suffer significant pain, fatigue, discomfort, morning stiffness and potentially irreversible disability in affected areas [10].

The ACR and EULAR guidelines for RA treatment recommend a ‘treat-to-target’ approach and set remission or alternatively low disease activity (LDA) in patients with long-standing disease as the treatment goal [11–13]. Despite the increased range of RA treatment options and available therapies to help slow disease progression and development of joint damage, it is estimated that most patients are not achieving remission, particularly in more established diseases [14–20]. Even patients achieving clinical remission (CR) can still experience radiographic progression [21]. Suboptimal management of RA can result in increased healthcare resource use and medical costs [18, 22] as well as poorer patient QoL [23].

Although remission is the aim, published studies report remission rates ranging from 5% to 45%, depending on the definition applied [24]. However, several studies have demonstrated that with intensive treatment around 60% of patients with early RA can reach sustained remission [25–27].

In Ireland, there is insufficient data on CR rates [28]. Despite ready access to biologic therapies, the Meteor international RA registry reported that Ireland had one of the poorest biological bDMARDs (bDMARDs) use/remission ratios amongst the 12 countries it studied. However, this study focused on bDMARDs use vs other DMARDs using data from two centres, limiting its representativeness of the national RA population [29, 30]. Likewise, other published retrospective data often derive from a single centre and focus on remission rates with bDMARDs [31].

This cross-sectional study intended to compare QoL outcomes for Irish RA patients considered to be responding to RA treatment (being in remission or LDA) vs those considered not responding (experiencing disease activity despite stable (>3 months) treatment). The study explored levels of RA disease activity, characteristics of patients in the responder and non-responder groups, and the impact of response/non-response on patient outcomes. Additionally, the economic impact of sub-optimally managed disease was explored. This data is expected to provide greater insight into the extent and impact in Ireland of unmet needs in RA.

## Materials and methods

### Study design

CONTExT-RA was a single-country, multicentre, cross-sectional, non-interventional study conducted in six rheumatology centres across Ireland. Patients were enrolled from March to November 2021 and attended a single routine study visit. All treatment decisions were per local standard of care and independent of study participation. Consecutive patients meeting the selection criteria were invited to participate. The Independent Ethics Committee or Institutional Review Board at each study site approved the study protocol, informed consent forms, and recruitment materials before patient enrolment. The studies were conducted in accordance with the International Conference for Harmonisation guidelines, applicable regulations and the Declaration of Helsinki. All patients provided written informed consent before screening. To calculate the sample size required to assess the primary endpoint and detect a statistically significant difference, the following assumptions for Clinical Disease Activity Index (CDAI) response rates were made: CR (10%), LDA (25%), moderate disease activity (MDA)/high disease activity (HDA) (65%), resulting in a sample size of 130 patients.

### Study population

Adult patients with a diagnosis of RA confirmed by a consultant rheumatologist in a hospital setting, were enrolled if being treated with an approved conventional/targeted synthetic (csDMARD/tsDMARD) or bDMARD and on a stable dose for at least 3 months. A maximum of 50% of enrolled patients could be treated with only csDMARD therapies. The patient population was stratified by CDAI score, with two levels of responders defined. Responder Group 1 (RG1) included patients in CR (CDAI score ≤2.8) only, and Responder Group 2 (RG2) included patients in CR and those with LDA (CDAI score >2.8–10). Non-responders (NRG) were defined as patients with MDA/HDA (CDAI >10). All patients who provided written informed consent were required to complete written in-clinic patient-reported outcome questionnaires. Additional data were collected through patient interviews and medical notes, including Health Resource Utilization (HRU), medical history, demography, treatment, planned changes to treatment, disease characteristics and predictors of response to treatment.

### Outcome measures

The primary endpoint was the EuroQol-5 dimensions (EQ-5D-5L) score in the responder groups compared with the NRG. EQ-5D-5L is a research instrument used to evaluate QoL, which consists of a descriptive system and the EQ-VAS. The descriptive system consists of five dimensions (mobility, self-care, usual activities, pain/discomfort and anxiety/depression), which patients rate using five levels to indicate their current health status. Patients use the EQ-VAS to self-rate their health status using a scale of 0 (worst imaginable) to 100 (best imaginable) [32]. Secondary endpoint outcome variables included joint pain measures (Visual Analogue Scale [VAS]) to assess pain [33], Functional Assessment of Chronic Illness Therapy-Fatigue Scale (FACIT-F) to assess fatigue [34], HAQ-Disability Index (HAQ-DI) to assess function [35], Work Productivity and Activity Impairment-Rheumatoid Arthritis (WPAI-RA) to assess productivity [36], and Healthcare Resource Utilization (HRU). Higher scores indicate worse outcomes for joint pain VAS, HAQ-DI and WPAI-RA, whilst lower scores for FACIT-F indicate worse outcomes. Supplementary Table S1, available at *Rheumatology Advances in Practice* online, provides more information on interpreting outcome measures.

### Statistical analysis

Three analysis sets were defined for the study. (1) The ‘Enrolled’ population included all patients who provided informed consent and fulfilled selection criteria. (2) The Full Analysis Set (FAS) population included all enrolled patients who had data recorded on the eCRF other than eligibility. All enrolled patients were included in the FAS, which was the main population used for analysis of primary and secondary endpoints. (3) The Safety Population was used for producing demographic and safety data tables. The Safety and FAS populations were identical.

The EQ-5D-5L index score, using Irish-based conversion metrics [37], was compared between response groups using a non-parametric Wilcoxon Rank-Sum test (5% significance level). Comparisons were performed in hierarchical order to control for type one error: Remission/LDA compared with MDA/HDA and remission compared with MDA/HDA. All secondary endpoints were stratified by response.

Further subgroup analyses for primary and secondary endpoints were performed according to RA treatment, disease activity (high vs moderate) and comorbidities.

Statistical analysis was conducted using SAS^®^ software version 9.4 (SAS Institute Inc., Cary, NC, USA).

## Results

### Patient demography and clinical characteristics

130 patients with a confirmed diagnosis of RA and receiving any approved DMARD therapy were included in the study. Using CDAI, 34 (26.2%) patients were assessed as being in CR, 42 (32.3%) LDA, 41 (31.5%) MDA and 13 (10.0%) HDA patients (Table 1).

**Table 1.** Disease activity assessment and responder group 1, responder group 2 and non-responder group populations

	Total (*N* = 130), *n* (%)
LDA (CDAI > 2.8–10)	42 (32.3)
HDA (CDAI > 22)	13 (10.0)
MDA (CDAI > 10–22)	41 (31.5)
CR (CDAI <= 2.8)	34 (26.2)
Responders	
1. CR	34 (26.2)
2. CR + LDA	76 (58.5)
Non-responders	
MDA + HDA	54 (41.5)

The mean (s.d.) age across the study cohort was 60.1 (13.36) years. MDA/HDA patients, classified as the non-responder group (NRG), had a mean age of 63.7 (13.00) years. This was higher than for patients in CR or in CR/LDA, classified as RG1 and RG2 respectively, who reported mean ages of 57.1 (12.91) and 57.6 (13.13) years. The majority (68.5%) of patients were female and white (95.4%) (Table 2).

**Table 2.** Demographics and sociodemographics for responder group 1, responder group 2 and non-responder group patients

	Responder group 1, CR (*N* = 34)	Responder group 2, CR+LDA (*N* = 76)	Non-responder group, MDA + HDA (*N* = 54)	Total (*N* = 130)
Age, years				
All, mean (s.d.)	57.1 (12.91)	57.6 (13.13)	63.7 (13.00)	60.1 (13.36)
Age group, *n* (%)				
18–30	0 (0.0)	2 (2.6)	1 (1.9)	3 (2.3)
31–40	5 (14.7)	8 (10.5)	3 (5.6)	11 (8.5)
41–50	3 (8.8)	12 (15.8)	5 (9.3)	17 (13.1)
>50	26 (76.5)	54 (71.1)	45 (83.3)	99 (76.2)
Gender, *n* (%)				
Female	23 (67.6)	51 (67.1)	38 (70.4)	89 (68.5)
Male	11 (32.4)	25 (32.9)	16 (29.6)	41 (31.5)
Race, *n* (%)				
Asian	1 (2.9)	2 (2.6)	2 (3.7)	4 (3.1)
Black	0 (0.0)	0 (0.0)	1 (1.9)	1 (0.8)
Mixed Race	0 (0.0)	1 (1.3)	0 (0.0)	1 (0.8)
White	33 (97.1)	73 (96.1)	51 (94.4)	124 (95.4)
Other	0 (0.0)	0 (0.0)	0 (0.0)	0 (0.0)
Duration of RA, years				
All, mean (s.d.)	11.2 (11.37)	11.3 (11.02)	12.1 (10.48)	11.6 (10.77)
Employment status, *n* (%)				
Employed full-time	14 (41.2)	26 (34.2)	8 (14.8)	34 (26.2)
Employed part-time due to RA	1 (2.9)	4 (5.3)	3 (5.6)	7 (5.4)
Employed part-time due to non-RA-related reasons	4 (11.8)	7 (9.2)	0 (0.0)	7 (5.4)
Unemployed due to RA and seeking work	1 (2.9)	1 (1.3)	1 (1.9)	2 (1.5)
Unemployed due to non-RA reasons and seeking work	0 (0.0)	1 (1.3)	2 (3.7)	3 (2.3)
Early retired because of RA	2 (5.9)	9 (11.8)	10 (18.5)	19 (14.6)
Early retired because of non-RA reasons	0 (0.0)	1 (1.3)	8 (14.8)	9 (6.9)
Regularly retired	8 (23.5)	20 (26.3)	15 (27.8)	35 (26.9)
Attending school or university	0 (0.0)	0 (0.0)	0 (0.0)	0 (0.0)
Other	4 (11.8)	7 (9.2)	7 (13.0)	14 (10.8)
Work productivity (during the past 7 days, how much did your RA affect your productivity while you were working? (Scale 0–10))				
All, mean (s.d.)	1.7 (2.52)	2.6 (2.78)	4.2 (3.28)	3.0 (2.96)
Level of education, *n* (%)				
Non-university, professional education	3 (8.8)	9 (11.8)	8 (14.8)	17 (13.1)
Primary school	7 (20.6)	14 (18.4)	16 (29.6)	30 (23.1)
Secondary school (e.g. high school)	16 (47.1)	35 (46.1)	24 (44.4)	59 (45.4)
University	8 (23.5)	18 (23.7)	6 (11.1)	24 (18.5)
No formal education	0 (0.0)	0 (0.0)	0 (0.0)	0 (0.0)
Place of living, *n* (%)				
Rural area <10 000 inhabitants	11 (32.4)	24 (31.6)	22 (40.7)	46 (35.4)
Towns 10 000–80 000 inhabitants	14 (41.2)	32 (42.1)	21 (38.9)	53 (40.8)
Urban centre >80 000 inhabitants	9 (26.5)	20 (26.3)	11 (20.4)	31 (23.8)
Smoking status, *n* (%)				
Never smoked	17 (50.0)	35 (46.1)	18 (33.3)	53 (40.8)
Smoker	7 (20.6)	12 (15.8)	12 (22.2)	24 (18.5)
Smoker pack years, mean (s.d.)	34.643 (31.0278)	35.375 (25.2309)	91.579 (223.882)	63.477 (158.431)
Ex-smoker	10 (29.4)	29 (38.2)	24 (44.4)	53 (40.8)
Ex-smoker pack years, mean (s.d.)	41.800 (31.7168)	38.448 (40.8522)	24.478 (19.3969)	32.269 (33.5804)
Alcohol units, per week				
All, mean (s.d.)	4.0 (5.98)	3.3 (6.31)	3.8 (6.05)	3.5 (6.19)

Overall, 97 patients (74.6%) had a positive test result for RF, and 89 (68.5%) had a positive test result for ACPA. Patients in CR reported the highest percentage (85.3% and 79.4%) compared with the NRG (74.1% and 63.0%), for RF/ACPA+ respectively.

A CRP test result was available for 129 patients. The highest mean (s.d.) level of 10.5 (27.33) was seen in the NRG, compared with 3.3 (3.77) and 4.3 (4.96) in RG1 and RG2, respectively (Supplementary Table S2, available at *Rheumatology Advances in Practice* online).

**Figure 1.**
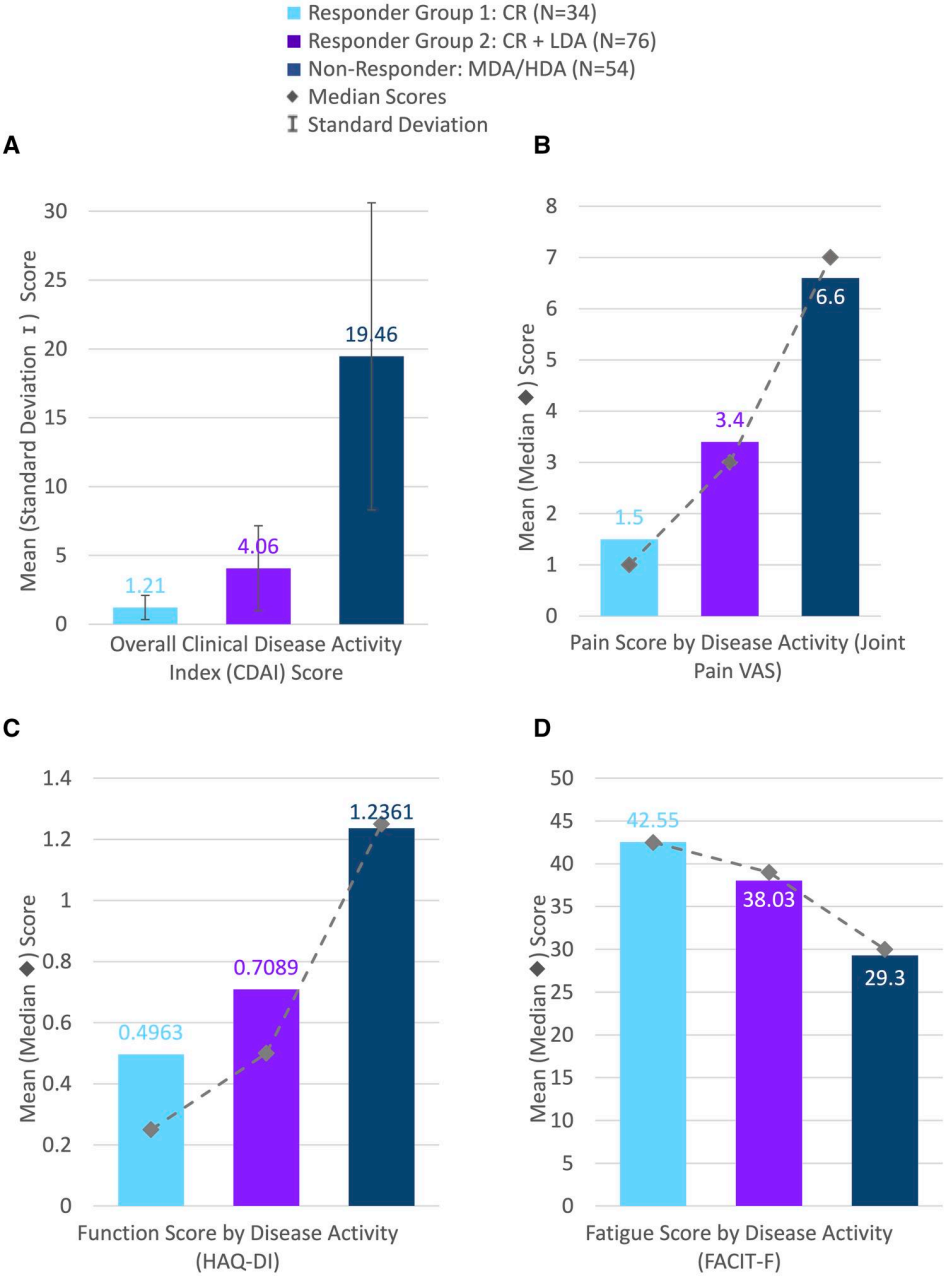
Disease activity and patient report outcome scores for responder and non-responder groups. (A) Mean scores for CDAI with standard deviation error bars. (B) Mean and median score for joint pain VAS. (C) Mean and median function score (HAQ-DI). (D) Mean and median fatigue score (FACIT-F). CDAI: Clinical Disease Activity; VAS: Visual Analogue Score; HAQ-DI: Health Assessment Questionnaire Disability Index; FACIT-F: Functional Assessment of Chronic Illness Therapy—Fatigue

For CDAI, the mean (s.d.) score for the NRG was 19.46 (11.162). Mean (s.d.) scores for RG1 and RG2 were much lower at 1.21 (0.883) and 4.06 (3.088), respectively (Fig. 1).

50.0% of RG1 patients had never smoked compared with 33.3% of the NRG. However, the proportion of current smokers was similar across groups (20.6%, 15.8% and 22.2% for RG1, RG2 and NRG, respectively).

More NRG patients (40.7%) reported living in a rural area than RG1 (32.4%) and RG2 (31.6%). RG1 and RG2 reported a higher education status than NRG, with 23.5% and 23.7% being university-educated compared with 11.1%, respectively. A higher proportion of the NRG (29.6%) selected primary school as their highest level of education, compared with RG1 (20.6%) and RG2 (18.4%).

Of non-responders, 18.5% retired early due to RA, compared with 5.9% and 11.8% in RG1 and RG2, respectively. Only 14.8% of non-responders were employed full-time, compared with 41.2% and 34.2% in RG1 and RG2, respectively. RG1 also reported significantly less impairment in recent (preceding week) work productivity than the NRG (Table 2).

90.7% of patients in the NRG reported at least one comorbidity, compared with 73.5% and 85.7% in CR and LDA, respectively. Cardiac disorder was the most common comorbidity, affecting 55.6% of non-responders compared with 35.3% and 26.2% of patients in CR and LDA, respectively. Additionally, the NRG had a higher proportion of patients with gastrointestinal disorders, psychiatric disorders, and vascular disorders (27.8%, 20.4% and 14.8%) compared with CR and LDA (11.8%, 11.8%, 2.9% and 4.8%, 9.5%, 7.1%, respectively).

116 patients received medication to treat comorbidities, including 25 patients in CR, 40 patients with LDA, and 51 patients with MDA/HDA (Supplementary Table S3, available at *Rheumatology Advances in Practice* online).

### Patient-reported outcomes

EQ-5D-5L index scores were significantly different in favour of RG1 (*P* < 0.001) when compared with the NRG. In summary, RG1 had a significantly higher QoL score, with a median EQ-5D-5L index of 0.920 (mean index score 0.866) compared with the NRG median index score of 0.629 (0.578). Additionally, the RG2 median index score of 0.822 (0.777), was also highly significantly different in favour of RG2 (*P* < 0.001) when compared with NRG (Table 3).

**Table 3.** Summary of EQ-5D-5L scores for responder group 1, responder group 2 and non-responder group

A				
	Responder group 1, CR (*N* = 34)	Non-responder Group, MDA + HDA (*N* = 54)	Total (*N* = 88)	*P*-value
*n*	34	53	87	<0.001^a^
Mean (s.d.)	0.8656 (0.14981)	0.5781 (0.26786)	0.6905 (0.26812)	
Median	0.9200	0.6290	0.7400	
Q1, Q3	0.7950, 1.0000	0.4810, 0.7610	0.5610, 0.9200	
Range	0.425, 1.000	−0.365, 0.932	−0.365, 1.000	
Missing	0	1	1	

B				
	Responder group 2, CR + LDA (*N* = 76)	Non-responder group, MDA + HDA (*N* = 54)	Total (*N* = 130)	*P*-value
*n*	76	53	129	<0.001^a^
Mean (s.d.)	0.7773 (0.20668)	0.5781 (0.26786)	0.6954 (0.25268)	
Median	0.8215	0.6290	0.7400	
Q1, Q3	0.6370, 0.9320	0.4810, 0.7610	0.5700, 0.9070	
Range	0.001, 1.000	−0.365, 0.932	−0.365, 1.000	
Missing	0	1	1	

a The *P*-value describes the statistical significance of the difference between the EQ-5D-5L scores between the two groups being compared.

A summary of patients’ worst joint pain (Fig. 1) demonstrated significantly different (*P* < 0.001) VAS scores in favour of the responder groups, with a median outcome score (mean index score) of 1 (1.5) and 3 (3.4), compared with the NRG with a score of 7 (6.6).

FACIT-F scores were also significantly different (*P* < 0.001) in favour of the responder groups, with a median outcome score (mean index score) of 42.5 (42.55) and 39 (38.03) for RG1 and RG2 respectively, in comparison to the NRG with a median score of 30 (29.3).

HAQ-DI scores were significantly different (*P* < 0.001) in favour of responder groups, with a median outcome score (mean index score) of 0.25 (0.4963) and 0.5 (0.7089) for RG1 and RG2 respectively, compared with the NRG median score of 1.25 (1.2361) (Fig. 1).

HRU is summarized in Supplementary Table S4, available at *Rheumatology Advances in Practice* online. For the NRG, 75.9% of patients reported at least one medical visit for their RA, in comparison to 55.9% and 51.3% of patients in RG1 and RG2, respectively.

### Treatment of RA: prior, current and next steps

**Figure 2.**
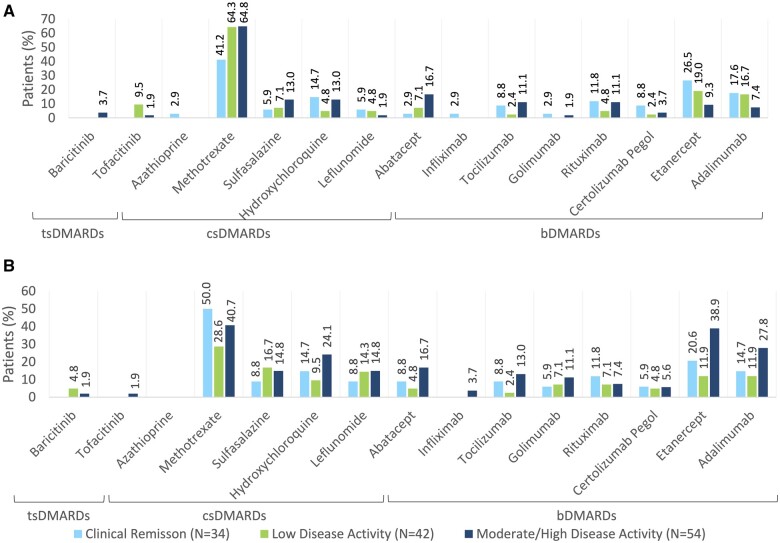
Treatments for RA and associated level of disease activity. (A) Current treatments. (B) Prior treatments. tsDMARDs: targeted synthetic DMARDs; csDMARDS: conventional synthetic DMARDs; bDMARDs: biological DMARDs

Current RA treatments are summarized in Fig. 2. While the study protocol stated, ‘a maximum of 50% csDMARD treated patients could be enrolled into the study’, only 40 patients (30.8%) were categorized as being in the csDMARD cohort. The most common treatment was MTX, with 58.5% of patients prescribed this csDMARD. Patients in CR had the lowest current use (41.2%), whereas use in the LDA and MDA/HDA was higher at 64.3% and 64.8% of patients, respectively.

A wide variety of biologic therapies were prescribed, the most common of which were etanercept, adalimumab and abatacept. Patients in MDA/HDA were less likely to be receiving the bDMARDs etanercept and adalimumab than patients in CR (9.3% and 7.4% vs 26.5% and 17.6% respectively), however were more likely to be receiving abatacept (16.7% *vs* 2.9% of RG1 patients).

A majority of 67 patients (51.5%) were receiving monotherapy, most commonly in LDA, with 27 (64.3%) patients compared with 17 (50.0%) and 23 (42.6%) patients in CR and MDA/HDA, respectively. Interestingly, whilst bDMARDs were the most common monotherapy, used in 41.2% of CR patients and 38 (29.2%) of all patients, a third (33.3%) of LDA patients were receiving csDMARD monotherapy as were almost a fifth (18.5%) of MDA/HDA patients. Only two (1.5%) patients, both in the LDA group, were treated with tsDMARD monotherapy (Table 4).

**Table 4.** Patients (%) in each disease activity group on DMARD monotherapy and combination therapy

	Clinical remission (*N* = 34)	Low disease activity (*N* = 42)	Moderate/high disease activity (*N* = 54)	Total (*N* = 130)
Combination therapy, *n* (%)	17 (50.0)	15 (35.7)	31 (57.4)	63 (48.5)
bDMARD/csDMARD	13 (38.2)	11 (26.2)	20 (37.0)	44 (33.8)
bDMARD/csDMARD/tsDMARD	1 (2.9)	0 (0.0)	0 (0.0)	1 (0.8)
csDMARD	3 (8.8)	2 (4.8)	8 (14.8)	13 (10.0)
csDMARD/tsDMARD	0 (0.0)	2 (4.8)	3 (5.6)	5 (3.8)
Monotherapy, *n* (%)	17 (50.0)	27 (64.3)	23 (42.6)	67 (51.5)
bDMARD	14 (41.2)	11 (26.2)	13 (24.1)	38 (29.2)
csDMARD	3 (8.8)	14 (33.3)	10 (18.5)	27 (20.8)
tsDMARD	0 (0.0)	2 (4.8)	0 (0.0)	2 (1.5)

Combination therapy was used in 63 (48.5%) patients with the most popular combination therapy involving a bDMARD and csDMARD, used in 38.2% of patients in CR and 37.0% in MDA/HDA.

Thirteen patients (10.0%) were treated with a csDMARD combination therapy—3 (8.8%), 2 (4.8%) and 8 (14.8%) patients in CR, LDA and MDA/HDA respectively.

Notably, only 6 (4.6%) patients received a tsDMARD as part of combination therapy, 1 (0.8%), 2 (1.5%) and 3 (2.3%) patients in CR, LDA and MDA/HDA, respectively.

One patient, in CR, was treated with three DMARDs.

Prior RA treatments are summarized in Fig. 2. Most patients received prior medication to treat RA (67.7%). Overall, 39.2% of patients recorded MTX as a prior RA treatment, including 50.0% of patients in CR, 28.6% of patients with LDA and 40.7% of patients in MDA/HDA. In the MDA/HDA group, abatacept, adalimumab and etanercept were reported as prior medications for 16.7%, 27.8% and 38.9% of patients, respectively.

Plans to switch/add a different DMARD were reported for 18 patients (13.8%) of whom 17 were in MDA/HDA (31.5% of all non-responders). Eleven (20.4%) MDA/HDA patients were planned to ‘switch’ treatment and 6 (11.1%) to ‘add’ a DMARD(s). Overall, the most common ‘switch’ was to a TNF inhibitor for 8 (14.8%) patients. The two most common ‘additions’ were a ‘conventional synthetic DMARD’ and rituximab with 2 (3.7%) patients intended for both (Supplementary Table S5, available at *Rheumatology Advances in Practice* online).

## Discussion

CONTExT-RA was a cross-sectional study evaluating disease control and associated disease burden, which reported that around a quarter (26.2%) of 130 patients treated in six secondary care rheumatology clinics across Ireland were assessed as being in CR for their RA. Furthermore, 41.5% of patients were assessed as having moderate or HDA. These outcomes fall well short of both the EULAR [12] and ACR [13] guidelines, which recommend CR as the primary therapeutic target, with LDA as an alternative goal in patients with long-standing disease. Unfortunately, despite the available therapies, such findings are commonly reported, with a recently published meta-analysis of 31 international studies involving 82 450 RA patients reporting pooled 3-, 6-, 12-, and 24-month remission rates of 17.2%, 16.3%, 21.5% and 23.5%, respectively [38].

Guidelines state it is not unusual for individual treatments to be ineffective, so it is considered best practice to treat towards a target of remission/LDA by switching between drugs sometimes as early as every 3 months if improvement in accordance with strategic principles is insufficient. Therefore, patients, rheumatologists and payers must be aware that multiple successive drug options are often needed to reach the therapeutic goal [12].

Surprisingly, only 17 (31.5%) poorly controlled patients were planned to change therapy and 15 (27.8%) did not report a prior treatment for RA. Additionally, a high percentage of the study cohort reported poor prognostic factors (positive RF and ACPA tests), which should result in earlier use of bDMARDs or tsDMARDs in combination with csDMARDs, according to EULAR treatment guidelines. Whilst almost two-thirds (64.8%) of patients in the NRG were receiving MTX, only a small majority of this cohort were receiving combination therapy (57.4%), and 33.3% were receiving csDMARDs only compared with 17.6% of patients in CR. Indeed, patients in CR were more likely to be receiving a bDMARD or tsDMARD (advanced therapies) than those with poor disease control (82.3% *vs* 66.7%).

In the NRG, abatacept, adalimumab and etanercept (bDMARDs) were reported as a prior medication for 16.7%, 27.8% and 38.9% of patients’, compared with current use of 16.7%, 7.4% and 9.3%, respectively. It is possible patients responded inadequately, or these medications were not well tolerated.

Given the number of patients with poor disease control, a relatively low uptake of tsDMARDs was observed, in just eight patients, including three in the NRG. While the reasons for treatment decisions including non-escalation of therapy in the NRG were not collected, contributing factors may include the relatively recent availability of the tsDMARDs for RA in Ireland, smoking history, and heightened awareness of cardiovascular/VTE risk with this class [39–42]. Comorbidities may also have played a part, particularly if concerns existed in relation to the potential for infection or pulmonary disease. This may explain the greater use of abatacept in this group, a biologic with, perhaps, a more favourable safety profile [43]. The higher mean age of the NRG may have influenced a lower prevalence of biologic prescriptions, as reported in prior publications [44].

As this study is a single ‘snap-shot’ in time for each patient, and the direction of disease progression is unknown, it is possible that current medication may have been recently prescribed or the condition is improving. However, given patients were on a stable dose of their current medication for at least 3 months and the typical visit frequency at sites, this seems unlikely.

Importantly, and supporting aiming for remission as a clear target, this study identified clear differences in EQ-5D-5L reported QoL between patients, depending on the level of disease activity. Patients in CR reported a relatively high mean EQ-5D-5L index score of 0.866, and a median index score of 0.920 comparable to the overall Irish population index referenced in literature, which reports 56.15% of the population scored 0.907 or more [44]. Given the study cohort’s relatively high age and published population norms elsewhere in Europe and the United States [45–47], it could be suggested QoL for RA patients in CR is maintained. For patients with moderate or HDA (NRG), the picture was significantly different with a mean EQ-5D-5L index score of 0.578 and a median score of 0.629. Consensus in the literature is that a minimally important difference in the EQ-5D-5L index score is between 0.04 and 0.1 [48, 49], much lower than the difference of almost 0.3 observed between the two groups in this study. The index score for patients with moderate and HDA is aligned with the bottom quartile of the Irish population [45]. The EQ-5D-5L index score for patients in remission and with LDA was around 0.1 lower than for those in remission alone, with a mean of 0.777 and a median of 0.822, suggesting overall QoL is lower in patients with LDA compared with those in remission but higher than those with moderate or HDA.

Differences in outcomes based on disease activity were also observed in joint pain VAS scores. Patients in CR reported a median score of 1 compared with 7 for those with moderate or HDA. Similarly, FACIT-F and HAQ-DI scores indicated patients in CR and with LDA reported significantly better outcomes.

This trend was also evident in the levels of HRU, with patients with moderate or HDA approximately 50% more likely to have attended a medical visit for their RA. Indeed, the patient-reported outcomes suggest the CDAI tool is an effective indicator of disease burden.

Allied with differences in patient-reported outcomes, other socio-demographic variations emerged. Patients with moderate or HDA were approximately six years older, had a lower level of formal education, were considerably less likely to be employed (14.8% vs 41.2% of patients in CR) and more likely to have retired earlier due to RA, although the latter two findings may be explained by the difference in age. These patients were also more likely to have smoked, live in rural areas, and have a comorbidity, with 55.6% having a cardiac comorbidity compared with 26.2% of LDA patients.

The study has some limitations often inherent in observational and cross-sectional studies, such as selection bias, restriction of conclusions to a single time-point and a limited sample size. However, the investigation includes a real-world cohort of patients across Ireland and captures detailed clinical and laboratory outcomes. Additionally, the requirement for stable DMARD therapy, ongoing for at least 3 months, provides for a better reflection of the treatment efficacy.

In summary, the results of this study suggest that in Ireland, disease control for many patients with RA treated in secondary care is not optimal and may disproportionately affect disadvantaged members of society. The observed impact of poor disease control is significant and wide ranging. With less than a third of poorly controlled patients planned to change treatment, there may be a sense that effective treatment options are limited or exhausted. However, outcomes for patients in CR clearly evidence the value of striving for greater disease control by adopting a more proactive ‘treat-to-target’ approach, reinforcing its benefits through education, and promoting the understanding that its success is dependent on prescribers performing additional disease activity assessments (e.g. CDAI scores) at frequent intervals according to a therapeutic protocol. Whilst current resources in rheumatology services may have been a limiting factor in this study, by successfully implementing ‘treat-to-target’, the burden of disease for patients, caregivers, physicians, the wider health system and economy may be greatly reduced.

## Supplementary Material

rkae132_Supplementary_Data

## Data Availability

AbbVie is committed to responsible data sharing regarding the clinical trials we sponsor. This includes access to anonymized, individual and trial-level data (analysis data sets), as well as other information (e.g. protocols, clinical study reports, or analysis plans), as long as the trials are not part of an ongoing or planned regulatory submission. This includes requests for clinical trial data for unlicensed products and indications. These clinical trial data can be requested by any qualified researchers who engage in rigorous, independent, scientific research and will be provided following review and approval of a research proposal, Statistical Analysis Plan (SAP), and execution of a Data Sharing Agreement (DSA). Data requests can be submitted at any time after approval in the United States and Europe and after acceptance of this manuscript for publication. The data will be accessible for 12 months, with possible extensions considered. For more information on the process or to submit a request, visit the following link: https://vivli.org/ourmember/abbvie/ then select ‘Home’.
