# Supplementary material for: CONTExT-RA: a cross-sectional study evaluating disease activity, quality of life and the socio-demographic profile of Irish patients with rheumatoid arthritis
Source: Rheumatol Adv Pract. 2024 Nov 6;8(4):rkae132. doi: 10.1093/rap/rkae132 (PMC11588026; doi:10.1093/rap/rkae132)
Supplement: rkae132_Supplementary_Data [file rkae132_supplementary_data.docx]

**SUPPLEMENTARY TABLE S1**: Additional information for outcome assessment measures.

| **Assessment** | **Abbreviation** | **Measurement** | **Interpretation** |
| --- | --- | --- | --- |
| EuroQol-5 dimensions-5 level Score | EQ-5D-5L | The descriptive system comprises five dimensions: mobility, self-care, usual activities, pain/discomfort and anxiety/depression.  Each dimension has five levels: no problems, slight problems, moderate problems, severe problems and extreme problems.  The patient is asked to indicate his/her health state by ticking the box next to the most appropriate statement in each of the five dimensions.  This decision results in a 1-digit number that expresses the level selected for that dimension. The digits for the five dimensions can be combined into a 5-digit number that describes the patient’s health state. The 5-digit number is then converted into a single-digit index values using Irish-based conversion metrics outlined in literature, with a maximum score of 1.0 (best possible health).  The EQ VAS records the patient’s self-rated health on a vertical visual analogue scale, where the endpoints are labelled ‘0 - The best health you can imagine’ and ‘100 - The worst health you can imagine’. | 1.0 is the best possible outcome. Lower scores indicate worse outcomes. |
| Joint Pain (Visual Analogue Scale) | Joint Pain VAS | Patients were asked to assess their level of joint pain over the last 7 days using a visual analogue scale. The scale ranged from ‘0 – No join pain’ to ’10 – worst possible join pain’. | Higher scores indicate worse outcomes |
| Functional Assessment of Chronic Illness Therapy – Fatigue Scale | FACIT-F | The FACIT-F scale is a validated self-report questionnaire, which asks patient’s to assess their level of fatigue during the usual daily activities over the past week.  It comprised of 13 questions which are each scored using a four-point Likert scale as follows: 0=Not At All; 1=A Little Bit; 2=Somewhat; 3=Quite A Bit; 4=Very Much. The sum of the individual item scores gives a result ranging from 0 – 52. | Lower scores indicate worse outcomes |
| Heath Questionnaire-Disability Index | HAQ-DI | The HAQ-DI instrument comprises of 20 questions over 8 categories. Patients were asked to rate each question on a scale of 0 – 3, where 0=without any difficulty; 1 = with some difficulty; 2 = with much difficulty or with assistance; 3 = unable to do.  The highest component score within each category was averaged to give a total disability score between 0 – 3. | Higher scores indicate worse outcomes |
| Work Productivity and Activity Impairment-Rheumatoid Arthritis | WPAI-RA | The WPAI-RA is a patient-reported qualitative assessment of presenteeism, absenteeism, and daily activity impairment due to a RA over the last 7 days.  As part of the assessment, patients were asked to consider how much their RA affected their work productivity on a 0 – 10 scale, where 0= RA had no effect on my work and 10= RA completely prevented me from working. | Higher scores indicate worse outcomes |
| Health Resource Utilisation | HRU | Health resource utilisation for the 12 months preceding the study inclusion was assessed as follows:   - Number of hospital inpatient days in the past 6 months. - Number of hospitalizations in the past 6 months - Number of sick leave days (in employed subjects only) in the past 6 months - Number of sick leaves (in employed subjects only) in the past 6 months - Number of outpatient visits to each kind of health care provider in the past 6 months   The 6-months data was multiplied by two to obtain the 12-month HRU. | Higher scores indicate worse outcomes |

**SUPPLEMENTARY TABLE S2**: Laboratory Test Results for RA in Patients in Responder Groups and Non-Responder Group

|  | **Responder Group 1, CR (N=34)** | **Responder Group 2, CR+LDA (N=76)** | **Non-Responder Group, MDA + HDA (N=54)** | **Total (N=130)** |
| --- | --- | --- | --- | --- |
| **Rheumatoid Factor Test Result, n (%)** | | | | |
| Positive | 29 (85.3) | 57 (75.0) | 40 (74.1) | 97 (74.6) |
| Negative | 4 (11.8) | 16 (21.1) | 10 (18.5) | 26 (20.0) |
| Not available | 1 (2.9) | 3 (3.9) | 4 (7.4) | 7 (5.4) |
| **Anti-Citrullinated Protein Antibody Test Result, n (%)** | | | | |
| Positive | 27 (79.4) | 55 (72.4) | 34 (63.0) | 89 (68.5) |
| Negative | 4 (11.8) | 15 (19.7) | 10 (18.5) | 25 (19.2) |
| Not available | 3 (8.8) | 6 (7.9) | 10 (18.5) | 16 (12.3) |
| **C-Reactive Protein Test Result (mg/l)** | | | | |
| n | 34 | 75 | 54 | 129 |
| Mean (SD) | 3.3 (3.77) | 4.3 (4.96) | 10.5 (27.33) | 6.9 (18.24) |
| Median | 2.1 | 2.6 | 2.9 | 2.7 |
| Q1,Q3 | 1.0, 3.1 | 1.0, 5.3 | 1.0, 8.9 | 1.0, 6.2 |
| Range | 0.3, 18 | 0.3, 30 | 0.3,186 | 0.3,186 |
| Missing | 0 | 1 | 0 | 1 |
| <=10mg/l, n (%) | 32 (94.1) | 67 (88.2) | 43 (79.6) | 110 (84.6) |
| >10mg/l^, n (%) | 2 (5.9) | 8 (10.5) | 11 (20.4) | 19 (14.6) |

**SUPPLEMENTARY TABLE S3:** Health Resource Utilisation Outcomes for patients in Responder Groups and Non-Responder Group

|  | **Responder Group 1, CR (N=34)** | **Responder Group 2, CR+LDA (N=76)** | **Non-Responder Group, MDA + HDA (N=54)** | **Total (N=130)** |
| --- | --- | --- | --- | --- |
| **In the past 6 months, has the subject been seen by a health care professional for their RA?, n (%)** | | | | |
| No | 0 (0.0) | 0 (0.0) | 1 (1.9) | 1 (0.8) |
| Yes | 19 (55.9) | 39 (51.3) | 41 (75.9) | 80 (61.5) |
| General Practitioner | 9 (26.5) | 20 (26.3) | 19 (35.2) | 39 (30.0) |
| Nurse Practitioner | 6 (17.6) | 17 (22.4) | 14 (25.9) | 31 (23.8) |
| Dermatologist | 0 (0.0) | 0 (0.0) | 0 (0.0) | 0 (0.0) |
| Gastroenterologist | 0 (0.0) | 0 (0.0) | 1 (1.9) | 1 (0.8) |
| Psychologist/Psychiatrist | 0 (0.0) | 0 (0.0) | 1 (1.9) | 1 (0.8) |
| Rheumatologist | 10 (29.4) | 22 (28.9) | 24 (44.4) | 46 (35.4) |
| Pharmacist | 7 (20.6) | 16 (21.1) | 13 (24.1) | 29 (22.3) |
| Ophthalmologist | 0 (0.0) | 0 (0.0) | 3 (5.6) | 3 (2.3) |
| Other | 2 (5.9) | 5 (6.6) | 6 (11.1) | 11 (8.5) |
| **In the past 6 months, has the subject been seen in the Emergency Department for their RA?, n (%)** | | | | |
| No | 18 (52.9) | 38 (50.0) | 40 (74.1) | 78 (60.0) |
| Yes | 1 (2.9) | 1 (1.3) | 1 (1.9) | 2 (1.5) |
| **In the past 6 months, has the subject been admitted to the hospital due to their RA?, n (%)** | | | | |
| No | 19 (55.9) | 39 (51.3) | 41 (75.9) | 80 (61.5) |
| Yes | 0 (0.0) | 0 (0.0) | 0 (0.0) | 0 (0.0) |
| **In the past 6 months, has the subject seen a HCP for any other (non-RA) medical condition?, n (%)** | | | | |
| No | 24 (70.6) | 45 (59.2) | 27 (50.0) | 72 (55.4) |
| Yes | 10 (29.4) | 31 (40.8) | 27 (50.0) | 58 (44.6) |
| General Practitioner | 5 (14.7) | 16 (21.1) | 19 (35.2) | 35 (26.9) |
| Nurse Practitioner | 2 (5.9) | 6 (7.9) | 2 (3.7) | 8 (6.2) |
| Dermatologist | 0 (0.0) | 0 (0.0) | 0 (0.0) | 0 (0.0) |
| Gastroenterologist | 0 (0.0) | 1 (1.3) | 5 (9.3) | 6 (4.6) |
| Psychologist/Psychiatrist | 0 (0.0) | 1 (1.3) | 0 (0.0) | 1 (0.8) |
| Rheumatologist | 0 (0.0) | 0 (0.0) | 0 (0.0) | 0 (0.0) |
| Pharmacist | 1 (2.9) | 6 (7.9) | 10 (18.5) | 16 (12.3) |
| Ophthalmologist | 0 (0.0) | 2 (2.6) | 3 (5.6) | 5 (3.8) |
| Other | 5 (14.7) | 15 (19.7) | 7 (13.0) | 22 (16.9) |

**SUPPLEMENTARY TABLE S4**: RA Treatment Strategy for Patients in Clinical Remission, Low Disease Activity and Moderate/High Disease Activity.

|  | **Clinical Remission (N=34)** | **Low-Disease Activity (N=42)** | **Moderate / High Disease Activity (N=54)** | **Total (N=130)** |
| --- | --- | --- | --- | --- |
| **Is the patient receiving any current medications used to treat RA? n (%)** | | | | |
| Yes | 34 (100.0) | 42 (100.0) | 54 (100.0) | 130 (100.0) |
| **Has the patient received any prior medications used to treat RA? n (%)** | | | | |
| Yes | 24 (70.6) | 25 (59.5) | 39 (72.2) | 88 (67.7) |
| No | 10 (29.4) | 17 (40.5) | 15 (27.8) | 42 (32.3) |
| **Is there a plan to add or switch to a different DMARD? n (%)** | | | | |
| Yes | 1 (2.9) | 0 (0.0) | 17 (31.5) | 18 (13.8) |
| No | 33 (97.1) | 42 (100.0) | 37 (68.5) | 112 (86.2) |
| **Switch, n (%)** | 1 (2.9) | 0 (0.0) | 11 (20.4) | 12 (9.2) |
| Biological DMARD, T-cell receptor inhibitor | 0 (0.0) | 0 (0.0) | 1 (1.9) | 1 (0.8) |
| Biological DMARD, TNF-inhibitor | 1 (2.9) | 0 (0.0) | 8 (14.8) | 9 (6.9) |
| Targeted synthetic DMARD, JAK- inhibitor | 0 (0.0) | 0 (0.0) | 2 (3.7) | 2 (1.5) |
| **Add, n (%)** | 0 (0.0) | 0 (0.0) | 6 (11.1) | 6 (4.6) |
| Biological DMARD, TNF-inhibitor | 0 (0.0) | 0 (0.0) | 1 (1.9) | 1 (0.8) |
| Conventional synthetic DMARD | 0 (0.0) | 0 (0.0) | 2 (3.7) | 2 (1.5) |
| Biological DMARD, B-cell inhibitor^a^ | 0 (0.0) | 0 (0.0) | 2 (3.7) | 2 (1.5) |
| Biological DMARD, T-cell receptor inhibitor | 0 (0.0) | 0 (0.0) | 1 (1.9) | 1 (0.8) |

^a^Rituximab was the only ‘Biological DMARD, B-cell inhibitor’ reported.

**SUPPLEMENTARY TABLE S5**: RA Treatment Strategy for Patients in Clinical Remission, Low Disease Activity and Moderate/High Disease Activity.

|  | **Clinical Remission (N=34)** | **Low-Disease Activity (N=42)** | **Moderate / High Disease Activity (N=54)** | **Total (N=130)** |
| --- | --- | --- | --- | --- |
| **Is the patient receiving any current medications used to treat RA? n (%)** | | | | |
| Yes | 34 (100.0) | 42 (100.0) | 54 (100.0) | 130 (100.0) |
| **Has the patient received any prior medications used to treat RA? n (%)** | | | | |
| Yes | 24 (70.6) | 25 (59.5) | 39 (72.2) | 88 (67.7) |
| No | 10 (29.4) | 17 (40.5) | 15 (27.8) | 42 (32.3) |
| **Is there a plan to add or switch to a different DMARD? n (%)** | | | | |
| Yes | 1 (2.9) | 0 (0.0) | 17 (31.5) | 18 (13.8) |
| No | 33 (97.1) | 42 (100.0) | 37 (68.5) | 112 (86.2) |
| **Switch, n (%)** | 1 (2.9) | 0 (0.0) | 11 (20.4) | 12 (9.2) |
| Biological DMARD, T-cell receptor inhibitor | 0 (0.0) | 0 (0.0) | 1 (1.9) | 1 (0.8) |
| Biological DMARD, TNF-inhibitor | 1 (2.9) | 0 (0.0) | 8 (14.8) | 9 (6.9) |
| Targeted synthetic DMARD, JAK- inhibitor | 0 (0.0) | 0 (0.0) | 2 (3.7) | 2 (1.5) |
| **Add, n (%)** | 0 (0.0) | 0 (0.0) | 6 (11.1) | 6 (4.6) |
| Biological DMARD, TNF-inhibitor | 0 (0.0) | 0 (0.0) | 1 (1.9) | 1 (0.8) |
| Conventional synthetic DMARD | 0 (0.0) | 0 (0.0) | 2 (3.7) | 2 (1.5) |
| Biological DMARD, B-cell inhibitor^a^ | 0 (0.0) | 0 (0.0) | 2 (3.7) | 2 (1.5) |
| Biological DMARD, T-cell receptor inhibitor | 0 (0.0) | 0 (0.0) | 1 (1.9) | 1 (0.8) |

^a^Rituximab was the only ‘Biological DMARD, B-cell inhibitor’ reported.
